# Exogenous Superoxide Dismutase: Action on Liver Oxidative Stress in Animals with Streptozotocin-Induced Diabetes

**DOI:** 10.1155/2011/754132

**Published:** 2011-03-13

**Authors:** Fábio Cangeri Di Naso, Alexandre Simões Dias, Marilene Porawski, Norma Anair Possa Marroni

**Affiliations:** ^1^Laboratory of Experimental Hepatology and Physiology, Hospital de Clínicas de Porto Alegre, Universidade Federal do Rio Grande do Sul, 90050-170 Porto Alegre, RS, Brazil; ^2^School of Physical Education, Physical Therapy Course, Federal University of Rio Grande do Sul 90690-200, Porto Alegre , RS, Brazil; ^3^Pontifícia Universidade Católica do Rio Grande do Sul (PUCRS), 91530-000 Porto Alegre, RS, Brazil; ^4^Universidade Luterana do Brasil, 92120-015 Canoas, RS, Brazil

## Abstract

*Aim*. To investigate the effects of exogenous antioxidant copper zinc superoxide dismutase (Cu/Zn SOD) on oxidative stress in the experimental model of diabetes mellitus (DM). *Methods*. Twenty eight male Wistar rats divided in four groups were used: control (CO), controls treated with SOD (CO + SOD), diabetics (DM), and diabetics treated with SOD (DM + SOD). SOD (orgotein, 13 mg/Kg body weight was administered. DM was induced by a single streptozotocin injection (i.p., 70 mg/kg), and 60 days later, we evaluated liver oxidative stress. *Results*. Liver lipoperoxidation was increased in the DM group and significantly decreased in the DM + SOD group. Nitrite and nitrate measures were reduced in the DM and increased in the DM + SOD group, while iNOS expression in the DM group was 32% greater than in the CO and 53% greater in the DM + SOD group than in the DM group (*P* < .01). P65 expression was 37% higher in the DM (*P* < .05), and there was no significant difference between the DM and DM + SOD groups. *Conclusion*. SOD treatment reduced liver oxidative stress in diabetic animals, even though it did not change NF*κ*B. SOD also increased NO, probably by the increased dismutation of the superoxide radical. The iNOS expression increase, which became even more evident after SOD administration.

## 1. Introduction

Diabetes *mellitus* (DM) is an endocrine-metabolic disorder of increasing incidence and clinical relevance, contributing to high morbidity and mortality rates [1]. Due to population aging, urbanization, increased prevalence of obesity, and physical inactivity, the number of individuals affected by DM is increasing in many parts of the world [2]. In view of this growing incidence, the study of the physiological routes of DM becomes crucial for the emergence of novel therapeutic procedures [3].

Four physiopathological pathways are involved and cause the chronic complications of the disease: the polyols pathway, protein kinase C activation, increase in the hexosamine flow rate, and the advanced glycation end-products (AGE) pathway. Although the disorder presents different routes of activation, oxidative stress (OE) is present in all the above-mentioned pathways.

Strategies to reduce the formation of superoxide anion (O^−^·) and thus oxidative stress are relevant to the treatment of DM [4]. The action of O^−^· scavengers is performed by a group of antioxidant enzymes called superoxide dismutases (SODs), which catalyze the dismutation of O^−^· into hydrogen peroxide (H_2_O_2_) and oxygen (O_2_) efficiently and specifically. Mammal tissues have 3 SODs isoforms: Cu/Zn superoxide dismutase (SOD1), Mn SOD (SOD2), and extracellular SOD (EC-SOD, or SOD3). SOD1 is a 32 kDA homodimer cell protein containing copper and zinc, and it is present in the cytosol, nucleus, peroxisomes, and mitochondrial membrane. Its primary function is to reduce the steady intracellular concentration of superoxide [5].

At the same time McCord and Fridovich were purifying SOD, another team of researchers purified orgotein [6], an anti-inflammatory compound identical to SOD. Orgotein is the generic name adapted by the USAN Council (U.S. Adopted Names Council) for Cu/Zn superoxide dismutase (SOD). This compound is obtained from bovine liver through a process involving thermal treatment, enzymatic digestion of other proteins, and purification of the homogenate through ionic exchange chromatography [7]. In the 1980s, approximately six million doses of SOD were administered to patients in Europe [8]. A number of clinical trials showed the effects of using orgotein in inflammatory disorders. Among these it is highlighted the use of orgotein in acute complications secondary to radiotherapy and intense radiation in the head, neck [9] and pelvis [10], in Peyronie's disease [11, 12], arthritis [7, 13, 14], bladder contracture [11, 15], multiple sclerosis [16], and hypertrophic scars, and cheloids [17].

The liver is the main organ of oxidative and detoxifying processes as well as free radical reactions; in many diseases, biomarkers of oxidative stress are elevated in the liver at an early stage. Because liver is subjected to ROS-mediated injury in diabetes, our experiments were performed to investigate the potential protective effects of SOD treatment on liver oxidative stress and p65 and iNOS expression in an experimental model of chronic hyperglycemia.

## 2. Materials and Methods

### 2.1. Animals and Experimental Protocol

The experimental protocol was according to the norms established by the Ethical Research Committee of Health of the Research and Postgraduate Group of the Hospital de Clínicas of Porto Alegre, as well as the recommendations of the *Principles for Research Involving Animals *(NAS). Twenty-eight male Wistar rats obtained from the breeding colony of the Basic Health Sciences Institute of the Federal University of Rio Grande do Sul (UFRGS) were used. Their mean weight was 200–300 grams (g) at the start of the trials. They were kept at a room with controlled temperature (22 ± 4°C) and at a 12 h light/dark cycle (light from 7 a.m to 7 p.m).

DM was induced by a single intraperitoneal (i.p.) injection of streptozotocin—STZ (Sigma *Chemical Company*, *St. Louis*, MO, USA) at a dose of 70 mg/Kg body weight [18]. STZ was dissolved in citrate sodium buffer (0.1 M, pH 4.5) and citric acid (0.1 M, pH 4.5) and administered in the left abdominal region about 10 min after dilution in buffer solution. The animals in the control group were given only sodium chloride (NaCl 0.9%) i.p. at the same volume of the buffer that was used to dissolve STZ.

Twenty-eight male Wistar rats were divided in four groups: control (CO), controls treated with SOD (CO + SOD), diabetics (DM), and diabetics treated with SOD (DM + SOD). Superoide dismutase (Ontosein-orgotein-, Lab. Tedea-Meiji Farma, S. A., Madrid-Spain) was used at a dose of 13 mg/Kg body weight for the last seven days of the trial and was administered subcutaneously as described elsewhere [19]. On day 60, the animals were sacrificed and their liver removed for posterior analyses. 

After 60 days of DM, the animals were deeply anesthetized and killed by exsanguination. Blood was withdrawn from the retro-orbital sinus and centrifuged for 15 minutes for glycemia determination, and the liver was removed in portions and frozen at −80°C for posterior measurements.

### 2.2. Biochemical Analyses and Oxidative Stress

#### 2.2.1. Glycemia

For glycemia determination the colorimetric enzymatic test (Kit ENZI-COLOR, Bio Diagnóstica) was used, in which a reagent was mixed with 20 *μ*L of the plasma sample and read in spectrophotometer (CARY 3E —UV-Visible Spectrophotometer Varian) with wavelength of 500 nm. Animals with blood glucose concentration above 250 mg/dL were considered as diabetics.

#### 2.2.2. Oxidative Stress

The liver was homogenized with 9 mL of phosphate buffer (KCL 140 mM, phosphate 20 mM pH 7.4) per tissue gram. Protein concentration in the liver homogenates was determined using bovine albumin solution as described by Lowry [20].

Liver peroxidation was determined by the thiobarbituric acid reactive substances (TBA-RS) assay [21].

For determination of antioxidant enzyme superoxide dismutase (SOD) we used the technique based on inhibition of adrenochrome formation in epinephrine autoxidation [22], and antioxidant enzyme catalase activity was determined by measuring the exponential disappearance of H_2_O_2_ at 240 nm and was expressed as pmoles/mg protein [23].

The determination of selenium-dependent glutathione peroxidase (GPx) was obtained through a method that consists in measuring NADPH oxidation by glutathione reductase [24].

### 2.3. Nitrite and Nitrate Measures

Total nitrites was measured by Griess's method. The samples were incubated with enzymatic cofactors (Tris 1 mol/L, pH 7.5; NADPH 0.02 mmol/L), glucose 6-phosphate, (G6P) 5 mmol/L, glucose 6-P dehydrogenase (G6PDH) 10 U/ml, and nitrate reductase (1.75 U/ml; Sigma, St. Louis, MO) for one hour to convert nitrate into nitrite at room temperature. Nitrites were determined by the reaction of samples with Griess's reagent (1% sulfanilamide, 0.1% naphthylenediamine 2.3 mL phosphoric acid 85%). Total tissue nitrite (initial nitrite plus the nitrite produced from sodium reduction) was estimated at 540 nm, from a standard curve of sodium nitrite (10^−8^ a 10^−3^ mol/L). The results were expressed as mmol/L [25].

#### 2.3.1. Western Blot

Protein extraction and Western blotting were performed as described elsewhere [26]. The membranes were incubated with anti-iNOS polyclonal antibody and specific anti-p65 antibody (Santa Cruz Biotechnology). Binding to the primary antibody was detected through rabbit anti-immunoglobulin bound to HRP (DAKO A/S, Glostrup, Denmark). Protein detection was performed by chemiluminescence using a commercial kit ECL (Amersham Pharmacia Biotech, Little Chalfont, Great-Britain) exposing the membrane to this commercial mixture for one minute. A cassette tape was subsequently introduced with developing film (Amersham Hyperfilm ECL, UK) for about 2 minutes. 

After washing the film, the bands were quantified by densitometry using program Scion Image 4.02 for Windows (Scion Corporation, Frederick, USA), with results being expressed in relation to control percentage (100%).

#### 2.3.2. Statistical Analysis

Data were presented as mean ± standard deviation (SD) and analyzed with the Statistical Package for the Social Sciences version 15.0 (SPSS-15.0). Variables were tested for normality by the Kolmogorov-Smirnov test. One-way analysis of variance (ANOVA) was used for inter-group differences. The student Newman-Keuls posttest was used for parametric variables, and Person's test was used for correlation of variables. The level of significance was 5% (*P* < .05).

## 3. Results

Exogenous SOD administration failed to reduce glycemia in diabetic rats. There was a significant decrease in animal weight in the DM group, and SOD treatment (DM + SOD) did not reverse this condition (Table 1). The DM group showed a significant increase in liver lipoperoxidation and treatment with exogenous SOD significantly reduced these values (Table 2). SOD and GPx activities were reduced in the DM group and the DM + SOD group showed an increase in enzymatic activities. Catalase activity was increased in the DM group and the treatment with SOD restored the activity values close to those of the CO group. Nitrite and nitrate measures were reduced in the DM group and were increased after treatment with SOD (Table 2). In addition, a positive correlation was found between SOD activity and high levels of nitrites and nitrates (*P* = .023; *r* = 0.565).

**Figure 1:**
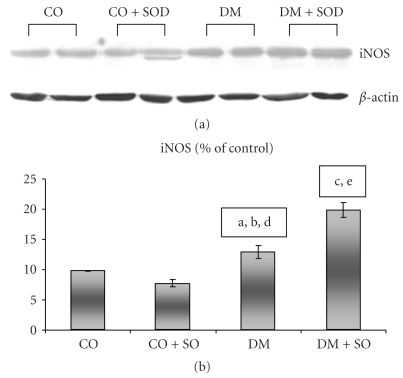
Effect of streptozotocin-induced diabetes and SOD on liver iNOS protein by Western blot analysis. Proteins were separated in SDS-polyacrylamide gel at 12% and incubated with anti-iNOS antibody. (a) CO versus DM *P* < .05; (b) DM versus DM + SOD *P* < .01; (c) CO + SOD versus DM + SOD *P* < .001; (d) CO + SOD versus DM *P* < .01; (e) CO + SOD versus DM + SOD *P* < .001.

**Figure 2:**
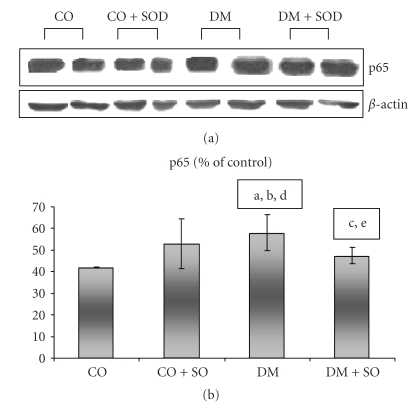
Effect of streptozotocin-induced diabetes and SOD on p65 protein by Western blot analysis. The nuclear fraction protein was separated in SDS-polyacrylamide gel at 12% and incubated with anti-p65 antibody. (a) CO versus DM *P* < .05; (b) DM versus DM + SOD *P* > .05; (c) CO + SOD versus DM + SOD *P* > .05; (d) CO + SOD versus DM *P* > .05; (e) CO + SOD versus DM + SOD *P* > .05.

INOS expression was increased in the DM group as compared to the CO group, and the SOD-treated group showed an increase as compared to the DM group itself (Figure 1). P65 had greater expression in the DM (*P* < .05) and no statistical difference as compared to the diabetic and SOD-treated groups (Figure 2).

## 4. Discussion

The results of the present study confirmed previous studies which described increased liver oxidative stress in the model of experimental diabetes mellitus [27]. These studies showed alterations in the activity of antioxidant enzymes associated with liver oxidative injury. Being rich in mitochondria to perform metabolic functions, the liver is a crucially important organ, and in a chronic hyperglycemic state the liver oxidative stress is considered a relevant process [28].

Exogenous SOD administration did not affect the glycemic values of diabetic animals. This finding contradicts a few results from previous studies which used antioxidants in the treatment of experimental DM [29]. Such different results could be due to the time and duration of the SOD treatment, which in our study occurred during the last week of a 60-day trial, whereas in other studies it was concomitant with induction.

SOD is an important antioxidant enzyme which rapidly catalyzes the dismutation of superoxide anion (O^−^·) and thus acts as a first line antioxidant defense. In the case of SOD deficiency or increased superoxide production, it reacts with nitric oxide to produce peroxynitrite (ONOO^−^), which is a potent oxidant and nitrosating agent that can cause direct damage to proteins, lipids, and DNA [8].

Since oxidative stress results from an imbalance between the generation of reactive oxygen species and/or nitrogen and the body's endogenous and exogenous defense ability, the study of the activity of antioxidant enzymes in DM becomes highly important.

In the present study, a significant increase was found in liver peroxidation in the DM group and a reduction in this oxidative stress marker following treatment with exogenous SOD, as well as reduced antioxidant enzymes SOD and GPx activities in DM and a significant increase after treatment. The reduction in SOD activity in the DM group can be explained by glycation in the EC-SOD heparin-binding domain. This domain anchors the protein to the endothelial cells surface and extracellular matrix of blood vessels [30, 31]. The affinity with heparin can be modulated by hyperglycemia, through the nonenzymatic glycation of EC-SOD lysine residues located in the heparin-binding domain. This glycation determines loss of heparin affinity but maintains SOD enzymatic activity in the extracellular medium. However, studies performed in diabetic patients show high serum levels of glycated EC-SOD and reduction in the arterial activity of this enzyme. These findings could be explained by glycation inhibiting the intracellular Cu/Zn SOD and this process indirectly affecting EC-SOD function [30, 31]. NO and its reactive species can cross the cell membrane and modulate the relaxation of smooth muscle and vascular tone response. In the extracellular medium, NO can establish a connection with great quantity of superoxide resulting from an extensive process of protein glycation in DM. Therefore, decreased vascular concentration of EC-SOD, reduced intracellular SOD activity, and increased production of superoxide anion are directly involved in the pathogenic process of DM, and the use of exogenous SOD may have a beneficial effect of reversing this process.

Peixoto et al. (2009) investigated the effects of a mimetic of SOD, the tempol, on renal oxidative stress in diabetic hypertensive rats. The study showed improvements in renal function accompanied by improvements in redox status with the use of exogenous enzyme. The improvement of the redox state can be proved by the increased expression of EC-SOD in renal cortex of animals treated with the SOD mimetic, in addition to reducing the generation of reactive oxygen species [32].

A number of studies have shown the involvement of NO and superoxide anion radical in the physiopathological process of chronic complications in DM [33]. Moreover, NO and other related free radicals and oxidative species are the greatest agents of *β*-pancreatic cell necrosis. In our study, there was evidence of a reduction in NO metabolites in the DM group and an increase in it after exogenous SOD administration. In addition, antioxidant enzyme SOD activity was found to be positively correlated with nitrite and nitrate levels (*P* = .023; *r* = 0.565). 

These findings show a greater availability of nitric oxide in diabetic animals treated with SOD, probably due to increased activity of antioxidant enzyme SOD. From these results one can establish a direct action from SOD dismutating the superoxide radical and an indirect action of this drug related to NO [9, 33]. Coppey et al. demonstrated the effect of treatment with a SOD functional mimetic M40403 to prevent vascular and neural complications in experimental diabetes. These studies provide additional evidence that diabetes-induced oxidative stress and the generation of superoxide and perhaps peroxynitrite may be partially responsible for the development of diabetic complications [34]. 

Through an imbalance in the redox state, DM triggers the activation of nuclear transcription factors such as NF*κ*B. The nuclear transcription factor NF*κ*B binds to DNA as a heterodimer of 50 kDa (p50) and 65 kDa (p65). The p65 subunit is responsible for the activation potential of NF*κ*B [35]. NF*κ*B activation is responsible for an increase in iNOS expression. In our study, as well as in Dias et al. [27], there was increased expression of nuclear p65 and iNOS, showing NF*κ*B activation in experimental DM. However, the use of exogenous SOD did not reduce p65 and increased iNOS expression. Experimental studies have demonstrated that the use of antioxidants can reduce oxidative stress, inhibit the activation of redox-dependent transcription factors [27], and the iNOS expression [36]. In our study, exogenous SOD administration determined changes to antioxidant enzyme activity and oxidative stress, but such changes did not cause p65 modification. There was iNOS expression increase, which became even more evident after SOD administration. The results of exogenous SOD administration in our experimental model differ from those of other *in vitro* studies showing iNOS regulation by antioxidant enzyme SOD [37, 38].

It is concluded that exogenous SOD treatment reduces liver oxidative stress in diabetic animals and increases antioxidant enzyme activity, despite not altering nuclear transcription factor NF*κ*B. Nevertheless, there is a significant change to p65 and iNOS, which justifies the performance of further studies in the use of exogenous antioxidants for DM treatment.

## Figures and Tables

**Table 1:** Changes in body weight and glycemia.

	*N*	Weight (g)	Glycemia (mg/dL)
CO	5	332.8 ± 27.66	237.95 ± 54.27
CO + SOD	6	312 ± 25.45	217.23 ± 27.45
DM	8	299.33 ± 46.17	481.29 ± 84.15
DM + SOD	9	266.28 ± 28.23	397.43 ± 29.15

Data appear as mean ± SD. CO: Control SOD: superoxide dismutase DM = Diabetes Mellitus *P* < .01—CO versus DM + SOD; CO + SOD versus DM + SOD *P* < .001—CO versus DM; CO + SOD versus DM.

**Table 2:** Oxidative stress, antioxidant enzyme activity and total nitrites and nitrates (NO_x_).

	CO (5)	CO + SOD (6)	DM (8)	DM + SOD (9)	ANOVA	
					CO versus DM	DM versusDM + SOD
TBARS (nmols/mg prot.)	0.20 ± 0.03	0.21 ± 0.04	0.36 ± 0.15	0.18 ± 0.04	*P* < .05	*P* < .01
SOD (U/mg prot.)	37.53 ± 11.61	39.83 ± 10.53	22.74 ± 4.23	62.73 ± 20.18	*P* < .05	*P* < .0001
CAT (pmols/mg prot.)	0.18 ± 0.02	0.21 ± 0.06	0.27 ± 0.03	0.17 ± 0.03	*P* < .01	*P* < .001
GPx (nmols/mg prot.)	0.91 ± 0.06	0.75 ± 0.19	0.53 ± 0.10	0.76 ± 0.16	*P* < .01	*P* < .05
NO_x_ (mM NO^3^+NO^2^)	2.32 ± 0.54	2.24 ± 0.27	1.47 ± 0.61	2.28 ± 0.20	*P* < .05	*P* < .05
